# Relationship between -889 C/T polymorphism in interleukin-1A gene and risk of chronic periodontitis: Evidence from a meta-analysis with new published findings

**DOI:** 10.4317/medoral.21233

**Published:** 2016-12-06

**Authors:** Felipe-Rodolfo-Pereira da Silva, Any-Carolina-Cardoso Guimarães-Vasconcelos, Luiz-Felipe de-Carvalho-França, David di-Lenardo, Luana-Silva Rodrigues, Maria-Luísa-Lima Barreto-do-Nascimento, Daniel-Fernando-Pereira Vasconcelos

**Affiliations:** 1 Laboratory of Histological Analysis and Prepare (LAPHIS), Federal University of Piaui, Parnaiba, Piaui, Brazil; 2 Doctorate in Biotechnology by the Northeast Biotechnology Network - RENORBIO, Teresina, Piaui, Brazil; 3 Post Graduation Program in Biomedical Sciences, Federal University of Piaui, Parnaiba, Piaui, Brazil; 4 Post Graduation Program in Dentistry, Federal University of Piaui, Teresina, Piaui, Brazil

## Abstract

**Background:**

Periodontitis results from an inflammatory response caused by accumulative microorganisms in periodontal sites. Several factors are involved in pathogenesis of periodontitis, for example the -889 C/T polymorphism in interleukin-1A gene. This study aimed to evaluate the relationship between this polymorphism and risk of development of chronic periodontitis by a meta-analysis based in new published findings.

**Material and Methods:**

Thereunto a review in literature was performed in the electronic biomedical and education databases (Cochrane Library, Google Scholar, MEDLINE and PubMed) to studies published before August 2, 2015, the abstracts were evaluated and the data extraction performed by two calibrated examiners. The calculations of the meta-analysis were obtained through statistical software Review Manager version 5.2 with calculation of Odds Ratio (OR), heterogeneity (I²) and Funnel plots with *P* <0.05.

**Results:**

In overall, twenty-one case/control studies were selected with 2,174 patients with chronic periodontitis and 1, 756 controls. The meta-analysis showed T allele was associated with chronic periodontitis (OR = 1.22, 95% CI: 1.09, 1.36, *P* = 0.0004) with decreased value to heterogeneity (I² = 15%, *P* = 0.28). TT genotype was associated to patients with chronic periodontitis (OR = 1.40, 95% CI: 1.07, 1.83, *P* = 0.01). No publication bias was found in this meta-analysis by asymmetry in Funnel plots.

**Conclusions:**

This meta-analysis with 2,174 patients with chronic periodontitis and 1, 756 controls evidenced the -889 C/T polymorphism is associated to risk of development of chronic periodontitis with no significant value to heterogeneity to allelic evaluation.

**Key words:**Alleles, odds ratio, periodontal disease, cytokines.

## Introduction

Periodontitis is a chronic inflammatory disease caused by accumulative plaque beyond gingival sulcus and host-immune response with involvement of multifactorial process (1). The disease receives various classifications being the most common: the aggressive periodontitis and chronic periodontitis.

In physiopathology of chronic periodontitis several inflammatory mediators contribute with damage in periodontal sites as well as connective tissue and alveolar bone loss (2). For example it has been interleukin-1 (IL-1) that has participative role in inflammatory process found in chronic periodontitis with its physiological variants: IL-1A, IL-1B and IL1 receptor antagonist.

IL-1A facilities and amplifies inflammatory response by induction of adhesion molecules to cellular infiltrate (3) and monocyte stimulation with bone resorption (4).

Genetic variations in IL-1A gene were associated with elevated risk to chronic periodontitis by elevated level of this cytokine in gingival fluid (5), but results are contradictory.

Three meta-analysis evaluating a polymorphism (-889 C/T) in IL-1A and chronic periodontitis are available in literature (6,7,8) and addressed association of this polymorphism and risk of development of chronic periodontitis. Meta-analysis is a statistical tool used by its capacity of to detect association between studies and annul the limited coverage of genetic variability that studies with small simple size bring.

However, since 2013 a significant number of studies have been published (9-13), these new data can bring others existing results in the literature. So, the aim of this study was perform a meta-analysis with recent findings which can clarify the relationship between -889 C/T polymorphism and the risk of development of chronic periodontitis.

## Material and Methods

- Data sources

A systematic search in literature was performed by three investigators in the electronic biomedical and education databases (Cochrane Library, Google Scholar, MEDLINE and PubMed) to studies published before August 2, 2015 and addressing the association of -889 C/T polymorphisms in IL-1A gene and risk of development of chronic periodontitis. The following combined keywords were used to retrieve the literature: (“interleukin” or “cytokine”) and (“polymorphism” or “-889 C/T” or “rs1800587”) and (“periodontitis” or “periodontal disease”). No language restriction was placed on the search and all citations of studies were screened to identify additional potential studies.

- Inclusion criteria

Articles were included in current meta-analysis if the studies met all the following criteria: [1] Evaluation of the polymorphism cited and risk of development of chronic periodontitis; [2] Studies are case/control design; [3] Genotype frequency documented; [4] Diagnosis of periodontitis confirmed through radiographic findings and clinical evaluation. Studies which did not bring sufficient information about genotype or allelic frequencies or did not respect any point these criteria were excluded.

- Data extraction

Two investigators independently reviewed all studies and extracted the data using a standardized form. Data were collected on the authors, year of publication, ethnicity, study design (case, control), number of cases and controls, age and subject type in study.

- Statistical analysis

The statistical analysis of data was performed with use of Review Manager version 5.2 software (RevMan, Nordic Cochrane Centre, The Cochrane Collaboration, 2012) and publication bias with Comprehensive Meta-analysis version 3.3.070 [2014] statistical software.

The chi-squared based Q statistic test (I²) was used to assess the presence of heterogeneity. When value of I² was not statistically significant (I²<50%, *P*>0.05) the Fixed-effect model was used to estimate the pooled Odds Ratio (OR). In other hand when heterogeneity was significant (I²>50%, *P*<0.05) the Random-effects model was used to OR calculation. Both methods the *P* value <0.05 was considered statistically significant. Begg’s test and Egger’s linear regression test (with *P*<0.05) were used to evaluate potential publications bias of reported associations with funnel plot asymmetry. All of data in studies were dichotomous data expressed as OR with 95% of confidence intervals (CI) to assess the association between polymorphism in IL-1A gene and periodontitis.

## Results

- Characteristics of eligible studies

**Figure 1:**
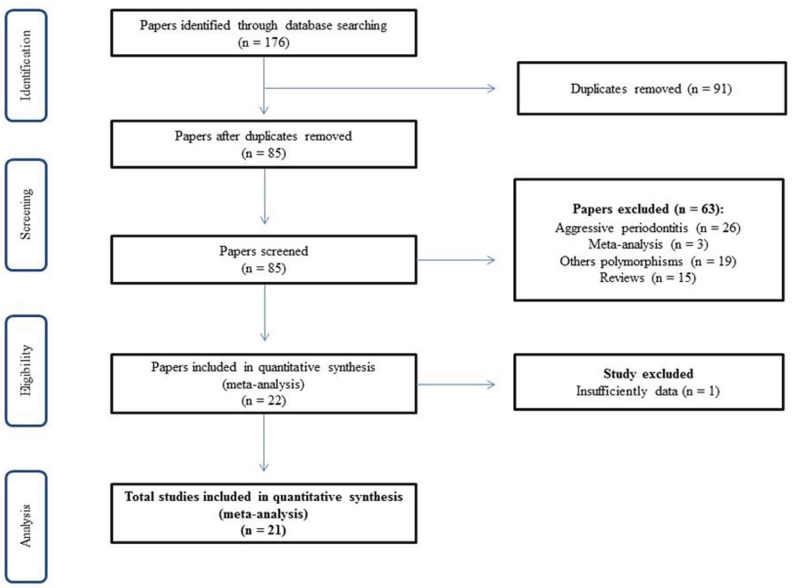
Flow diagram for inclusion of studies in meta-analysis.

Twenty-one case-control studies (9-29) were identified at finish of search in literature, included in this synthesis meta-analysis (Fig. 1). The studies were published at interval of 1998 to 2015. In overall, this current meta-analysis included 2,174 patients with chronic periodontitis and 1,756 controls from various ethnic groups ( Table 1). Fourteen studies were carried out in Caucasian, five in Asian and two in mixed population. Three studies performed an evaluation stratifying the individuals in smokers and non-smokers.

**Table 1:**
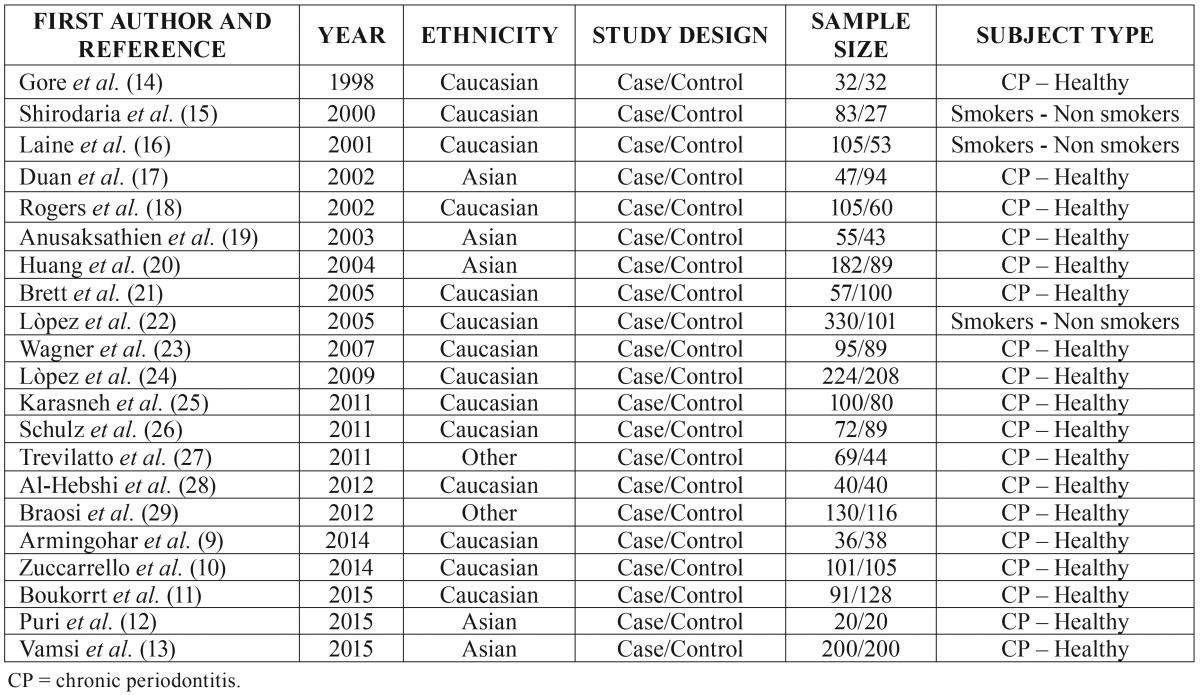
Baseline characteristics of studies included in this current meta-analysis.

- Statistical results

**Figure 2:**
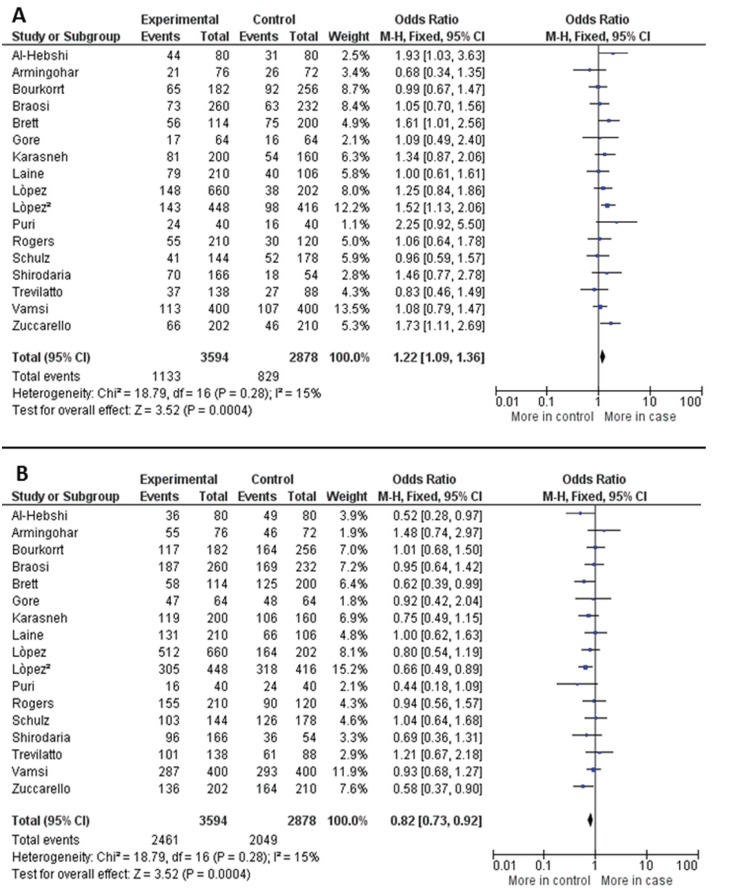
A. Forest plot of comparison of T allele in -899 polmorphism in IL-1A gene and risk of chronic periodontitis. B. Forest plot of comparison of C allele in -899 polymorphism in IL-1A gene and risk of chronic periodontitis.

The meta-analysis showed the -889 C/T in IL-1A gene is associated to elevated risk development of chronic periodontitis. In allelic evaluation, four studies (17,19,20,23) caused elevated heterogeneity (I² = 71%, *P*<0.00001) and were outlier in funnel plot graphic, after exclusion, the heterogeneity decreased to be unremarkable (I² = 15%, *P* = 0.28), although these studies did not carry out heterogeneity in genotypic evaluation. The forest plots to T allele versus C allele in overall analysis and to C allele versus T allele were shown in figure 2A and 2B, respectively. T allele was significantly associated to patients case (OR = 1.22, 95% CI: 1.09, 1.36, *P* = 0.0004) and C allele was associated to control group (OR = 0.82, 95% CI: 0.73, 0.92, *P* = 0.0004). In both calculations the Fixed-effect statistical model was used to estimate the pooled Odds Ratio. Besides, TT genotype was associated to patients with chronic periodontitis in overall (OR = 1.40, 95% CI: 1.07, 1.83, *P* = 0.01). The  Table 2 brings all genetic calculated models as well as the stratified analysis by ethnicity, smokers and non-smokers, and by sex.  Table 3 containing data about heterogeneity in all calculated models.

**Table 2:**
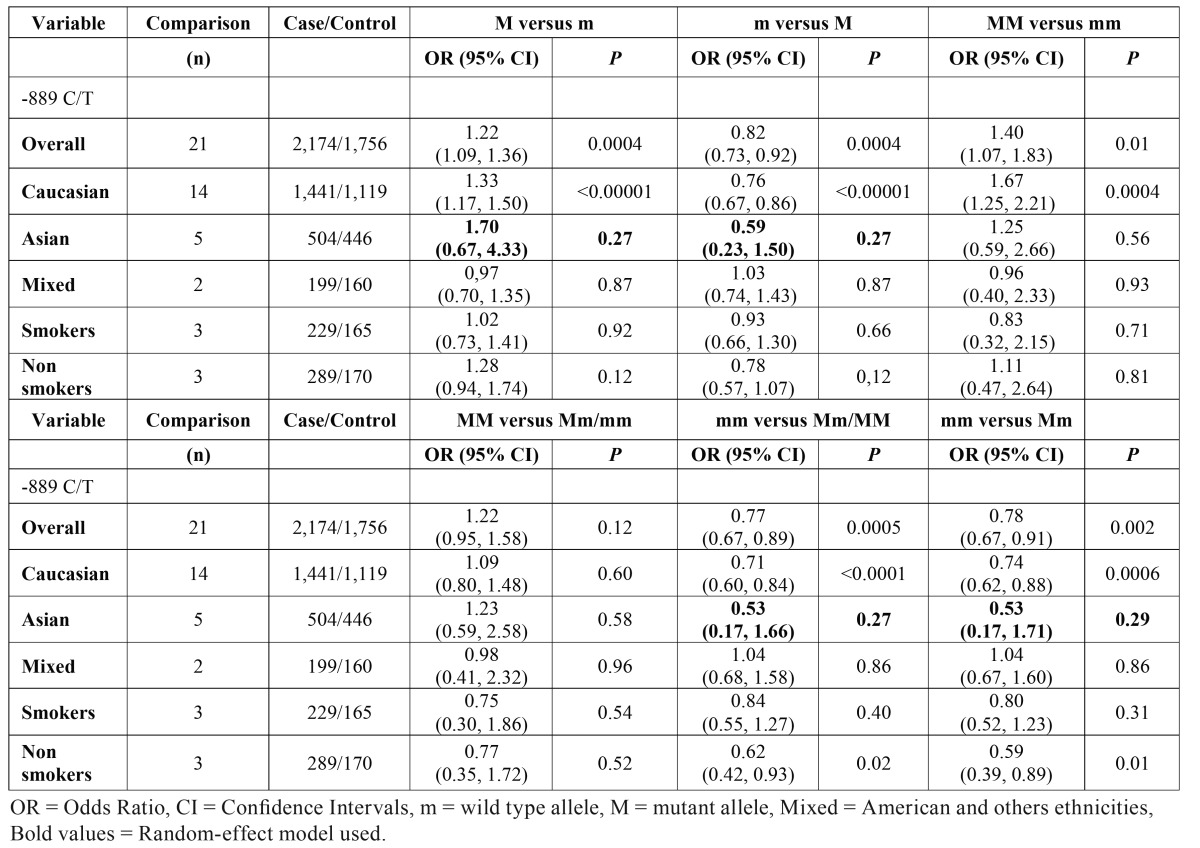
Meta-analysis of -889 C/T polymorphism in IL-1A gene and risk of chronic periodontitis (allelic and genotypic models).

**Table 3:**
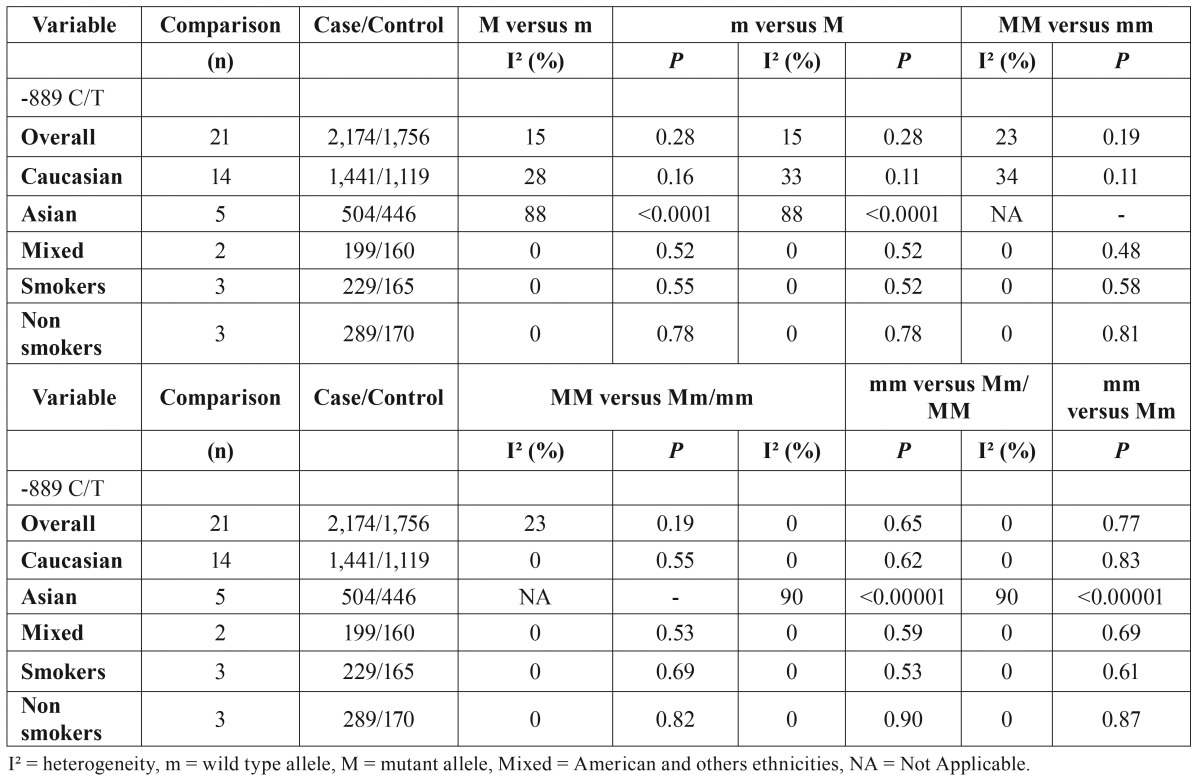
Value of heterogeneity to all allelic and genotypic models in current meta-analysis.

- Sensitivity analysis and Publication bias

**Figure 3:**
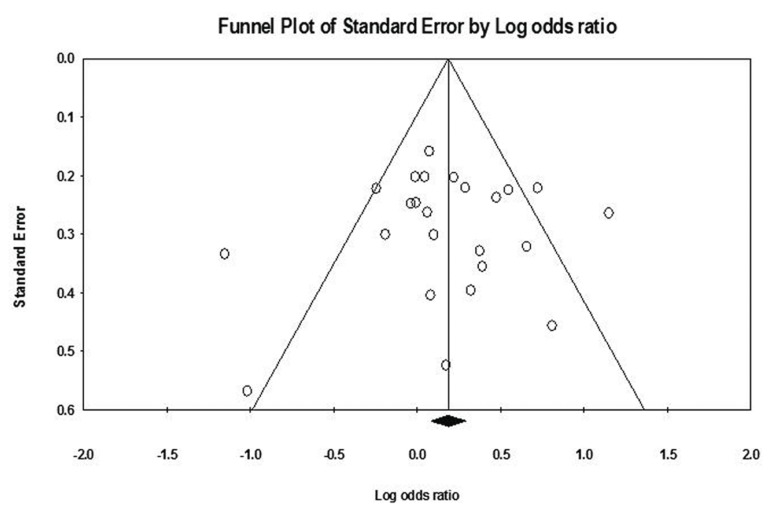
Funnel plot to publication bias in meta-analysis about-899 C/T polymorphism in IL-1A gene and risk of chronic periodontitis.

To evaluate the individual effect of studies a sensitivity analysis was performed by omitting each study to assess this impact on pooled ORs. No single publication changed the pooled ORs qualitatively, which suggested that results of this meta-analysis were accurate. The Begg’s test and Egger’s linear regression test did not reveal any indication of publication bias in allelic evaluation (*P* = 0.901 and *P* = 0.791, respectively) as showed by no funnel plot asymmetry in figure 3.

## Discussion

Interleukin-1A is a soluble molecule participant in host-response against microbial agents with guiding inflammatory cells into infection sites, stimulation of monocytes and bone resorption (5). In an experiment in which lysate cell were injected into the peritoneal cavity of mice in a model of peritonitis, an inflammatory response and neutrophil infiltration occurred in an IL-1A dependent manner (30). As an interleukin which modulates immune response by action on monocytes, variation in its gene could predispose to several inflammatory processes during periodontitis.

It has been reported several genetic variant in IL-1A gene associated to periodontitis but results are inconsistence.

The meta-analysis was performed to evaluate the change C allele to T allele in -889 position on this gene and demonstrated this polymorphism is associated to elevated risk in development of chronic periodontitis in overall evaluation, with no significant value of heterogeneity ( Table 3). These findings could be explained by new studies published since two years ago.

Zuccarelo *et al.* (10) demonstrated no association between their results about -889 C/T polymorphism in IL-1A gene in chronic periodontitis when compared to previous published studies by Comparison of the Carriage-rate of the Rare Allele (CRA). When compared by meta-analysis, the data were combined with several others results increasing the power of association.

-889 C/T polymorphism in this cytokine also was reported in other inflammatory process such as development of irritant contact dermatitis (31). Significant association between this polymorphism and elevated levels of IL-1A in localized aggressive periodontitis was identified (32) but not risk of lung cancer in Chinese population (TT genotype - OR = 0.809, 95% CI: 0.18, 3.56, *P* = 0.779) (33).

In stratified evaluation on ethnicity, the T allele was associated to risk of development of chronic periodontitis in Caucasian group (OR = 1.33, 95% CI: 1.17, 1.50, *P*<0.00001). This finding contradicts others studies carried out in Caucasian populations from Australia (18) and Algerian (11).

A previous meta-analysis evaluated the -889 C/T polymorphism in adult whites with chronic periodontitis showed this genetic variation was associated to the disease (7), however the results from this study are inconsistent because elevated heterogeneity. Heterogeneity proves how these studies are inconsistent, important fact to meta-analysis because the presence or absence of true heterogeneity can affect the statistical model applied on data (34).

Although a study demonstrated there was not association between TT or CC genotypes with patients when compared with controls in a Norwegian population (9), these two genotypes both were associated to patients with chronic periodontitis in this meta-analysis ( Table 2).

This polymorphism was descripted in association with higher frequency in patients with chronic periodontitis (20). In evaluation on Asian ethnicity, the results indicated -889 C/T polymorphism was not associated with chronic periodontitis in all models calculated ( Table 2). These data could be biased by elevated heterogeneity ( Table 3) and use of Random-effects as statistical model in meta-analysis to stratified evaluation.

In mixed population, the meta-analysis did not demonstrate significant association between -889 C/T polymorphism in IL-1A gene and risk of chronic periodontitis in T versus C evaluation (OR = 0.97, 95% CI: 0.70, 1.35, *P* = 0.87) corroborating data previously found in Brazilian population (27). The racial variation in Brazilian can explain the different results published in other study about this polymorphism (29).

The evaluation about this polymorphism in smokers and no smokers was performed and indicated no association with chronic periodontitis. Nevertheless, a limited number of studies (15,18,22) in this separated analysis may represent a bias in meta-analysis. Meisel *et al.* (35) suggested a linkage between extents of periodontitis with smokers which carried out positive genotype for this polymorphism in IL-1A gene. Moreover in other study the smoking was proved as cause of increased risk of attachment loss independently of IL-1A genotype and the interaction between genotype and smoking status caused elevated risk of periodontitis (36).

Our data demonstrate how the T allele predisposes the development of chronic periodontitis, such data becomes more significant when comparing it with a study on the same polymorphism, -889 C/T polymorphism in IL-1A, to a patient with peri-implantitis (*P* = 0.024), which showed an OR = 10.9 for individuals that presented previous periodontitis (37). The association of these information together with others (6-8,38,39) should provide an alert for clinical planning for surgery implants in individuals with previous history of periodontitis.

Although these results are robust and this meta-analysis is the first to evaluate only this polymorphism; the meta-analysis had some limitations. First, in Asian population, the meta-analysis was interfered by elevated heterogeneity had been used the Random-effect as statistical model. The Type 1 error could be causing the results found.

Second, the articles published after the last identified meta-analysis in literature brought elevated impact to assess the association between this polymorphism and periodontitis decreasing heterogeneity; however more studies are necessary to conclude the influence of -889 C/T polymorphism in IL-1A gene in chronic periodontitis, especially about gender evaluation.

In conclusion, this meta-analysis, composed by twenty-one studies in various ethnic groups totaling 2,174 patients with chronic periodontitis and 1,756 controls, showed T allele in -889 C/T was associated to risk of development of chronic periodontitis (OR = 1.22, 95% CI: 1.09, 1.36, *P* = 0.0004) and C allele was associated to control group (OR = 0.82, 95% CI: 0.73, 0.92, *P* = 0.0004), both in overall analysis.

## References

[1] Teng HC, Lee CH, Hung HC, Tsai CC, Chang YY, Yang YH (2003). Lifestyle and psychosocial factors associated with chronic periodontitis in Taiwanese adults. J Periodontol.

[2] Agrawal AA, Kapley A, Yeltiwar RK, Purohit HJ (2006). Assessment of single nucleotide polymorphism at IL-1A+ 4845 and IL-1B+ 3954 as genetic susceptibility test for chronic periodontitis in Maharashtrian ethnicity. J Periodontol.

[3] Graves DT, Cochran D (2003). The contribution of interleukin-1 and tumor necrosis factor to periodontal tissue destruction. J Periodontol.

[4] Maney P, Owens JL (2015). Interleukin polymorphisms in aggressive periodontitis: A literature review. J Indian Soc Periodontol.

[5] Quappe L, Jara L, López NJ (2004). Association of interleukin-1 polymorphisms with aggressive periodontitis. J Periodontol.

[6] Nikolopoulos GK, Dimou NL, Hamodrakas SJ, Bagos PG (2008). Cytokine gene polymorphisms in periodontal disease: A meta-analysis of 53 studies including 4178 cases and 4590 controls. J Clin Periodontol.

[7] Karimbux NY, Saraiya VM, Elangovan S, Allareddy V, Kinnunen T, Kornman KS (2012). Interleukin-1 gene polymorphisms and chronic periodontitis in adult whites: a systematic review and meta-analysis. J Periodontol.

[8] Mao M, Zeng XT, Ma T, He W, Zhang C, Zhou J (2013). Interleukin-1α -899 (+4845) C→T polymorphism increases the risk of chronic periodontitis: evidence from a meta-analysis of 23 case-control studies. Gene.

[9] Armingohar Z, Jørgensen JJ, Kristoffersen AK, Schenck K, Dembic Z (2014). Polymorphisms in the Interleukin-1 Gene Locus and Chronic Periodontitis in Patients with Atherosclerotic and Aortic Aneurysmal Vascular Diseases. Scand J Immunol.

[10] Zuccarello D, Bazzato MF, Ferlin A, Pengo M, Frigo AC, Favero G (2014). Role of familiarity versus interleukin-1 genes cluster polymorphisms in chronic periodontitis. Gene.

[11] Boukortt KN, Saidi-Ouahrani N, Boukerzaza B, Ouhaibi-Djellouli H, Hachmaoui K, Benaissa FZ (2015). Association analysis of the IL-1 gene cluster polymorphisms with aggressive and chronic periodontitis in the Algerian population. Arch Oral Biol.

[12] Puri K, Chhokra M, Dodwad V, Puri N (2015). Association of interleukin-1 α (-889) gene polymorphism in patients with generalized aggressive and chronic periodontitis. Dent Res J (Isfahan).

[13] Lavu V, Venkatesan V, Venkata Kameswara Subrahmanya Lakkakula B, Venugopal P, Paul SF, Rao SR (2015). Polymorphic regions in the interleukin-1 gene and susceptibility to chronic periodontitis: a genetic association study. Genet Test Mol Biomarkers.

[14] Gore EA, Sanders JJ, Pandey JP, Palesch Y, Galbraith GM (1998). Interleukin-1beta+3953 allele 2: Association with disease status in adult periodontitis. J Clin Periodontol.

[15] Shirodaria S, Smith J, McKay IJ, Kennett CN, Hughes FJ (2000). Polymorphisms in the IL-1A gene are correlated with levels of interleukin-1α protein in gingival crevicular fluid of teeth with severe periodontal disease. J Dent Res.

[16] Laine ML, Farré MA, González G, van Dijk LJ, Ham AJ, Winkel EG (2001). Polymorphisms of the interleukin-1 gene family, oral microbial pathogens, and smoking in adult periodontitis. J Dent Res.

[17] Duan H, Zhang J, Zhang Y (2002). The association between IL-1 gene polymorphisms and susceptibility to severe periodontitis (in Chinese). Hua Xi Kou Qiang Yi Xue Za Zhi.

[18] Rogers MA, Figliomeni L, Baluchova K, Tan AE, Davies G, Henry PJ (2002). Do interleukin-1 polymorphisms predict the development of periodontitis or the success of dental implants? J Periodontal Res.

[19] Anusaksathien O, Sukboon A, Sitthiphong P, Teanpaisan R (2003). Distribution of interleukin-1β+ 3954 and IL-1α-889 genetic variations in a Thai population group. J Periodontol.

[20] Huang HY, Zhang JC (2004). Investigation on the association of interleukin-1 genotype polymorphism with chronic periodontitis (in Chinese). Hua Xi Kou Qiang Yi Xue Za Zhi.

[21] Brett PM, Zygogianni P, Griffiths GS, Tomaz M, Parkar M, D'Aiuto F (2005). Functional gene polymorphisms in aggressive and chronic periodontitis. J Dent Res.

[22] López NJ, Jara L, Valenzuela CY (2005). Association of interleukin-1 polymorphisms with periodontal disease. J Periodontol.

[23] Wagner J, Kaminski WE, Aslanidis C, Moder D, Hiller KA, Christgau M (2007). Prevalence of OPG and IL-1 gene polymorphisms in chronic periodontitis. J Clin Periodontol.

[24] López NJ, Valenzuela CY, Jara L (2009). Interleukin-1 gene cluster polymorphisms associated with periodontal disease in type 2 diabetes. J Periodontol.

[25] Karasneh JA, Ababneh KT, Taha AH, Al-Abbadi MS, Ollier WER (2011). Investigation of the interleukin-1 gene cluster polymorphisms in Jordanian patients with chronic and aggressive periodontitis. Arch Oral Biol.

[26] Schulz S, Stein JM, Altermann W, Klapproth J, Zimmermann U, Reichert Y (2011). Single nucleotide polymorphisms in interleukin-1gene cluster and subgingival colonization with Aggregatibacter actinomycetemcomitans in patients with aggressive periodontitis. Hum Immunol.

[27] Trevilatto PC, de Souza Pardo AP, Scarel-Caminaga RM, de Brito RB Jr, Alvim-Pereira F, Alvim-Pereira CC (2011). Association of IL1 gene polymorphisms with chronic periodontitis in Brazilians. Arch Oral Biol.

[28] Al-Hebshi NN, Shamsan AA, Al-Ak'hali MS (2012). Interleukin-1 Two-Locus Haplotype Is Strongly Associated with Severe Chronic Periodontitis among Yemenis. Mol Biol Int.

[29] Braosi AP, de Souza CM, Luczyszyn SM, Dirschnabel AJ, Claudino M, Olandoski M (2012). Analysis of IL1 gene polymorphisms and transcript levels in periodontal and chronic kidney disease. Cytokine.

[30] Eigenbrod T, Park JH, Harder J, Iwakura Y, Nunez G (2008). Cutting edge: critical role for mesothelial cells in necrosis-induced inflammation through the recognition of IL-1 alpha released from dying cells. J Immunol.

[31] Landeck L, Visser M, Kezic S, John SM (2012). IL1A-889 C/T gene polymorphism in irritant contact dermatitis. J Eur Acad Dermatol Venereol.

[32] Havemose-Poulsen A, Sørensen LK, Bendtzen K, Holmstrup P (2007). Polymorphisms within the IL-1 gene cluster: effects on cytokine profiles in peripheral blood and whole blood cell cultures of patients with aggressive periodontitis, juvenile idiopathic arthritis, and rheumatoid arthritis. J Periodontol.

[33] Bai L, Yu H, Wang H, Su H, Zhao J, Zhao Y (2013). Genetic single-nucleotide polymorphisms of inflammation-related factors associated with risk of lung cancer. Med Oncol.

[34] Higgins JP, Thompson SG, Deeks JJ, Altman DG (2003). Measuring inconsistency in meta-analyses. BMJ.

[35] Meisel P, Siegemund A, Dombrowa S, Sawaf H, Fanghaenel J, Kocher T (2002). Smoking and polymorphisms of the interleukin-1 gene cluster (IL-1β, IL-1α, and IL-1RN) in patients with periodontal disease. J Periodontol.

[36] Meisel P, Schwahn C, Gesch D, Bernhardt O, John U, Kocher T (2004). Dose-effect relation of smoking and the interleukin-1 gene polymorphism in periodontal disease. J Periodontol.

[37] García-Delaney C, Sánchez-Garcés MÁ, Figueiredo R, Sánchez-Torres A, Gay-Escoda C (2015). Clinical significance of interleukin-1 genotype in smoking patients as a predictor of peri-implantitis: A case-control study. Med Oral Patol Oral Cir Bucal.

[38] Chambrone L, Ascarza A, Guerrero ME, Pannuti C, de la Rosa M, Salinas-Prieto E (2014). Association of -1082 interleukin-10 gene polymorphism in Peruvian adults with chronic periodontitis. Med Oral Patol Oral Cir Bucal.

[39] Vasconcelos DF, da Silva MA, Marques MR, de Brito Júnior RB, Vasconcelos AC, Barros SP (2012). Lymphotoxin-Alpha Gene Polymorphism +252A/G (rs909253, A/G) Is Associated with Susceptibility to Chronic Periodontitis: A Pilot Study. ISRN Dent.

